# Could the gut microbiota community in the coral trout *Plectropomus leopardus* (Lacepède, 1802) be affected by antibiotic bath administration?

**DOI:** 10.1002/vms3.267

**Published:** 2020-04-19

**Authors:** Xing Zheng, Shengjie Zhou, Jing Hu, Rui Yang, Zhifeng Gu, Jian G. Qin, Zhenhua Ma, Gang Yu

**Affiliations:** ^1^ Tropical Aquaculture Research and Development Center South China Sea Fisheries Research Institute Chinese Academy of Fishery Sciences Sanya China; ^2^ Ocean College Hainan University Haikou P. R. China; ^3^ Key Laboratory of South China Sea Fishery Resources Exploitation and Utilization Ministry of Agriculture Guangzhou P. R. China; ^4^ College of Science and Engineering Flinders University Adelaide SA Australia

**Keywords:** bath administration, fluoroquinolone antibiotic, gut microbiota, *Plectropomus leopardus*

## Abstract

Gut microbiota in fish plays an important role in the nutrient digestion, immune responses and disease resistance. To understand the effect of fluoroquinolone antibiotic bath administration on fish gut microbiota, the gut microbiota community in the coral trout *Plectropomus leopardus* (Lacepède, 1802) was studied after enrofloxacin bathing treatment at two concentrations (5 and 10 mg/L) and 0 mg/L as control. A total of 90 fish were used in this study, and three replicates were used for each treatment. After a 24‐hr bath, the gut bacterial composition was analyzed using high‐throughput Illumina sequencing. The results indicated that the richness, diversity and the dominant bacterial taxa of *P. leopardus* gut bacteria were not affected by enrofloxacin bathing (*p* > .05). *Proteobacteria* and *Firmicutes* were the dominant phyla, and *Exiguobacterium*, *Citrobacter*, *Vibrio*, *Acinetobacter*, *Pseudomonas* were the dominant genus. The findings in the present study provide an understanding on the relationship between fish gut bacteria community and antibiotic bath administration. The findings of this study are instructive on the antibiotic bath administration applied for the management of *P. leopardus* health in aquaculture.

## Graphical abstract

The gut microbiota community in the coral trout *Plectropomus leopardus* (Lacepède, 1802) was studied after enrofloxacin bathing treatment at two concentrations (5 and 10 mg/L) and 0 mg/L as control. After a 24‐hr bath, the gut bacterial composition was analyzed using high‐throughput Illumina sequencing. The results indicated that the richness, diversity and the dominant bacterial taxa of *P. leopardus* gut bacteria were not affected by enrofloxacin bathing (*p* > .05).

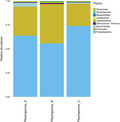

## 1 INTRODUCTION

The coral trout *Plectropomus leopardus* (Lacepède, 1802) is distributed from the Western Pacific to East Africa and the Red Sea (Yoseda et al., 2008). As a commercially important tropical marine fish species, artificial breeding and rearing of coral trout have been rapidly developed (Zhao et al., 2016). In China, the coral trout is widely farmed along the southern coast in the tropical and subtropical regions and maintains a high price at the fish market (Ma, Zhang, Guo, Zheng, & Zhang, 2015). However, farmed coral trout are sensitive to pathogens in stressful conditions such as high stocking density and poor water quality. Especially within the summer months, from July to September, coral trout aquaculture in Hainan Province in China suffers serious economic losses due to disease outbreaks on a regular annual basis (Gu et al., 2015; Yao et al., 2015). In order to prevent disease outbreaks, antibiotics, vaccines and immunostimulants are commonly employed on fish farms (Zhou et al., 2015).

It is well known that viral, bacterial and fungal diseases present a serious challenge to maricultural enterprises and there is a need to develop effective methods for disease control (Buchmann, 2015). In aquaculture, antibiotics and chemicals have traditionally been used to control fish diseases, particularly in hatcheries where larval fish are vulnerable to bacterial pathogens (Rico, & Van den Brink, 2014). Bacterial diseases are primarily controlled using three treatment routes: oral, intramuscular or bath administration. Bath administration is most commonly utilized due to convenience and ease of use (Fang, Zhou, & Liu, 2018).

The control of microbiota is essential in aquaculture; however, the administration of a medicine via bathing may impact on the health of aquatic animals through changes in the gut microbiota (Gilbert & Dupont, 2011; Mekuchi et al., 2018; Nayak, 2010; Xia et al., 2014). Gut microbiota, composed of a diverse and vast population of microorganisms, plays an important role in nutrient digestion, immune responses and disease resistance of fish (Austin, 2006; Gilbert & Dupont, 2011; Ray, Ghosh, & Ringø, 2012). Its composition and interactions affect the energy extraction efficiency and is important in the metabolism, immune system modulation (Moore, Christian, Sommer, & Gautam, 2011; Tremaroli & Bäckhed, 2012). In comparison to mammals, the fish gut microbiota composition is more likely to be affected by the environment (Xia et al., 2014). Community composition of gut microbes can be altered by many factors, such as stress (Paul, 2010) and nutritional status (Turnbaugh et al., 2009). An altered microbiota in the intestine can change host immune function and increase the risk of disease (Morgan et al., 2012). Despite the effectiveness of antibiotic bath treatment to control and prevent bacterial disease in fish, little is known about the impact of antibiotic bathing on gut microbiota of the host. Thus, it is important to elucidate the relationship between host gut microbiota composition and antibiotic bath treatment, for successful and efficient production of fish.

In the present study, the coral trout *P. leopardus* gut microbiota were characterized at different enrofloxacin concentrations. The aim of this study is to understand the effect of antibiotic bath treatment on the gut microbiota community of the coral trout. The findings of this study will improve our understanding of the relationship between fish gut bacteria and the amount of antibiotic application in aquaculture, would be instructive to health management and improve the safety and quality of seafood products.

## 2 MATERIALS AND METHODS

### 2.1 Animals and treatment

The experimental procedure was complied with the standards of Institutional Animal Care and Use Committee guidelines (Suckow & Lamberti, 2017). All experiments were conducted in line with the principles and guidelines for the care and use of live fish and the guidelines for animal experimentation approved by the Animal Experimental Council (AEC/NRIFS) of the National Research Institute of Fisheries Science, Fisheries Research Agency.

Prior to the trial, all fish were reared in an aquarium, supplied continuously with recirculating seawater (34 psu, 31°C) and dissolved oxygen by an air compressor (7.80 mg/L). They were fed with a commercial pelleted diet twice daily (TZU‐Feng Aquaculture Supplies Co., Ltd.).

Before the start of the experiment, fish were deprived of food for 24 hr and then 10 fish (body weight 120 ± 12 g) were randomly stocked into each of the nine 300‐L tanks containing 0, 5 and 10 mg/L enrofloxacin in triplicate. Throughout the experimental period, temperature, salinity and DO (dissolved oxygen) were maintained at 31°C, 34 psu, 7.20 mg/L respectively.

### 2.2 Enrofloxacin solution preparation

The formulation of enrofloxacin hydrochloride (CAS:93106‐60‐6, Solarbio Life Sciences Co., Ltd) for bath administration was dissolved into filtered fresh seawater with the addition of either 5 or 10 mg/L of enrofloxacin. The enrofloxacin concentration in water was then determined according to a previously reported HPLC method (Fang, Liu, Liu, & Lu, 2012).

### 2.3 Experimental design and sampling

At the start of the trial, fish were within one of three groups: Plectropomus A (control) in water containing 0 mg/L enrofloxacin, Plectropomus B in 5 mg/L enrofloxacin solution or Plectropomus C in 10 mg/L enrofloxacin solution. Each group was replicated in triplicate and remained within the bath solution for 24 hr. There were 10 fish in each tank and so there were 30 fish used in each group.

Approximately 10 fish from each experimental tank were randomly collected and euthanized using an overdose of tricaine methanesulfonate (MS‐222, Sigma‐Aldrich Pty Ltd.). The fish and then dissected with sterile scissors. The gut was filled with chyme were carefully collected and preserved into 1.5 ml sterile centrifuge tubes. All samples were stored at −80°C until DNA extraction was conducted.

### 2.4 DNA extraction

Samples were prepared for genomic DNA extraction using EZNA Stool DNA Kit (Omega Bio‐tek), according to the manufacturer's instructions. DNA concentrations and quality were checked using Qubit 3.0 Fluorometer (Invitrogen) and agarose gel electrophoresis.

### 2.5 Amplicon generation and library preparation

The purified DNA (20–30 ng) was used to generate amplicons. The V3 and V4 hypervariable regions of prokaryotic 16S rDNA were selected for generating amplicons and taxonomic analysis. A panel of proprietary primers were designed to detect the V3 and V4 variable regions in bacteria and Archaea16S rDNA.

The V3‐V4 region of bacteria 16S ribosomal RNA genes was amplified by PCR using the forward primers containing the sequence ‘CCTACGGRRBGCASCAGKVRVGAAT’ and reverse primers containing the sequence ‘GGACTACNVGGGTWTCTAATCC’. At the same time, indexed adapters were added to the ends of the 16S rDNA amplicons to generate indexed libraries ready for downstream NGS sequencing on Illumina Miseq.

PCR amplifications were performed in triplicate in a 25‐μl mixture containing 2.5 μl TransStart Buffer, 2 μl dNTPs, 1 μl of each primer and 20 ng template DNA. The thermal cycling program was performed as follows: 94°C for 3 min, followed by 24 cycles at 94°C for 5 s, 57°C for 90 s, 72°C for 10 s and a final extension at 72°C for 5 min. The PCR products were examined using 1.5% agarose gel, and then excised and purified using the QIAquick Gel extraction kit (Qiagen) according to the manufacturer's protocol.

### 2.6 Bacterial 16s rRNA gene sequencing and analyses

The purified PCR products were used for library preparation and high‐throughput sequencing. DNA library concentrations were validated by Qubit3.0 Fluorometer. To quantify the library to 10 nM, DNA libraries were multiplexed and loaded on an Illumina MiSeq instrument according to manufacturer's instructions (Illumina). Sequencing was performed using PE250/300 paired‐end, image analysis and base calling were conducted by the MiSeq Control Software (MCS) embedded in the MiSeq instrument.

The QIIME data analysis package was used for 16S rRNA data analysis (Caporaso et al., 2010). After sequencing, the paired‐end reads (forward and reverse reads) were joined, assigned to samples based on barcode and truncated by cutting off the barcode and primer sequence (Schloss et al., 2009). The quality filtering on joined sequences was performed and the sequences that did not fulfill the following criteria were discarded: sequence length <200 bp, no ambiguous bases and mean quality score ≥ 20. The sequences were then compared with the reference database (RDP Gold database) using the UCHIME (a unique sequence analysis tool) algorithm to detect chimeric sequences, and then the chimeric sequences were removed.

The effective sequences were used in the final analysis. Sequences were grouped into operational taxonomic units (OTUs) using the clustering program VSEARCH (1.9.6) against the Silva 128 database pre‐clustered at 97% sequence identity. The Ribosomal Database Program (RDP) classifier was used to assign taxonomic category to all OTUs at a confidence threshold of 0.8. The RDP classifier used the Silva 128 database that has taxonomic categories predicted to the species level (Caporaso et al., 2010).

Sequences were rarefied prior to calculation of alpha and beta diversity statistics. Alpha diversity indexes were calculated in QIIME from rarefied samples using the Shannon and Simpson index for measuring diversity and the ACE and Chao index for richness. Rarefaction curves were analyzed with MOTHUR (version.1.30). Beta diversity was calculated using principal coordinate analysis (PcoA) through ‘R vegan package’ (Lozupone & Knight, 2005). Heatmap was analyzed through ‘R vegan package’ using weighted unifrac distances (Kang et al., 2013). These data were analysed from each of the enrofloxacin concentrations (0, 5 and 10 mg/L) respectively.

### 2.7 Statistical analyses

The results were analysed by an unpaired Student's *T*‐test using SPSS version 18.0 (SPSSInc). Results were considered statistically significant when probability (*p*) values were less than .05.

## 3 RESULTS

### 3.1 Sequence analysis

**FIGURE 1:**
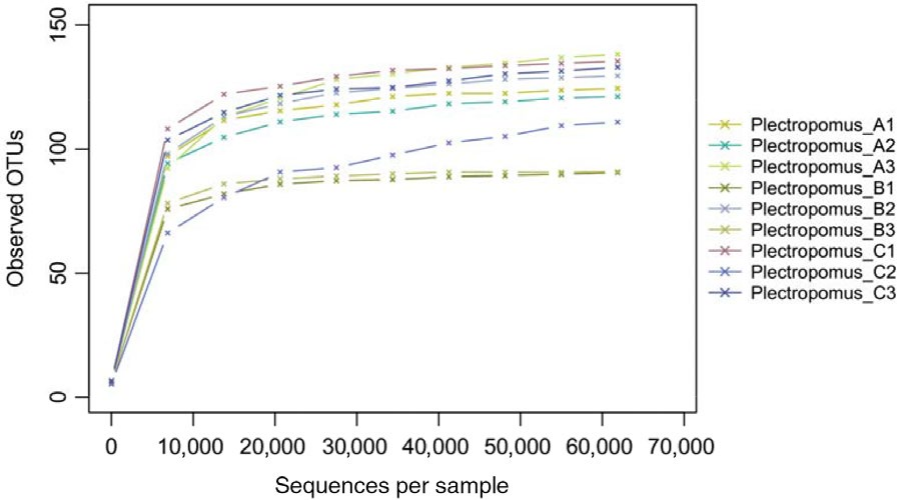
Rarefaction analyses of all samples in the control and enrofloxacin bathing treatment for coral trout *Plectropomus leopardus*. Rarefaction curves represent the number of operational taxonomic unit (OTU) detected in Plectropomus_A (A1, A2, A3), Plectropomus_B (B1, B2, B3) and Plectropomus_C (C1, C2, C3). Sequences were clustered at 97％ sequence similarity. Plectropomus_A = control group (no enrofloxacin), Plectropomus_B = 5 mg/L enrofloxacin bathing group, Plectropomus_C = 10 mg/L enrofloxacin bathing group

A total of 782,959 effective tags were obtained from all samples, with 427 bp average length. 1764 OTUs (Operational Taxonomic Units) were defined based on a similarity greater than 97% with average of 285 OTUs in each sample. Rarefaction curves indicated that the obtained sequence could reflect majority of the bacterial diversity in each sample (Figure 1). The estimators of community richness (ACE and Chao) and diversity (Shannon) are shown in Table 1, and there was no difference (*p* > .05). Results from the analysis of alpha diversity metrics showed that the microbial richness and diversity were not altered after enrofloxacin bathing (*p* > .05), even thought there was reduction in OUT’s, ACE and Chao indices.

**TABLE 1:** ACE, Chao, Shannon, Simpson and Good's coverage indices for 16s rRNA libraries of all samples from control and enrofloxacin bathing treatment for coral trout *Plectropomus leopardus*

Index	0 mg/L	5 mg/L	10 mg/L
ACE	131.78 ± 9.40	105.21 ± 22.97	129.99 ± 2.59
Chao	134.50 ± 11.91	104.92 ± 22.39	129.75 ± 16.20
Shannon	3.63 ± 0.24	3.42 ± 0.15	3.61 ± 0.58
Simpson	0.85 ± 0.03	0.83 ± 0.01	0.85 ± 0.05
Good's coverage	0.96 ± 0.08	0.94 ± 0.23	0.95 ± 0.13

Note

No significant difference was detected between the control and treatment groups (*p* > .05). The ACE calculator returns the ACE richness estimate for an OUT (Operational Taxonomic Units) definition (https://www.mothur.org/wiki/Ace). The Chao calculator returns the Chao1 richness estimate for an OTU definition (https://www.mothur.org/wiki/Chao). The Shannon calculator returns the Shannon diversity index for an OTU definition (https://www.mothur.org/wiki/Shannon). The Simpson calculator returns the Simpson diversity index for an OTU definition (https://www.mothur.org/wiki/Simpson).

### 3.2 Taxonomic composition

**FIGURE 2:**
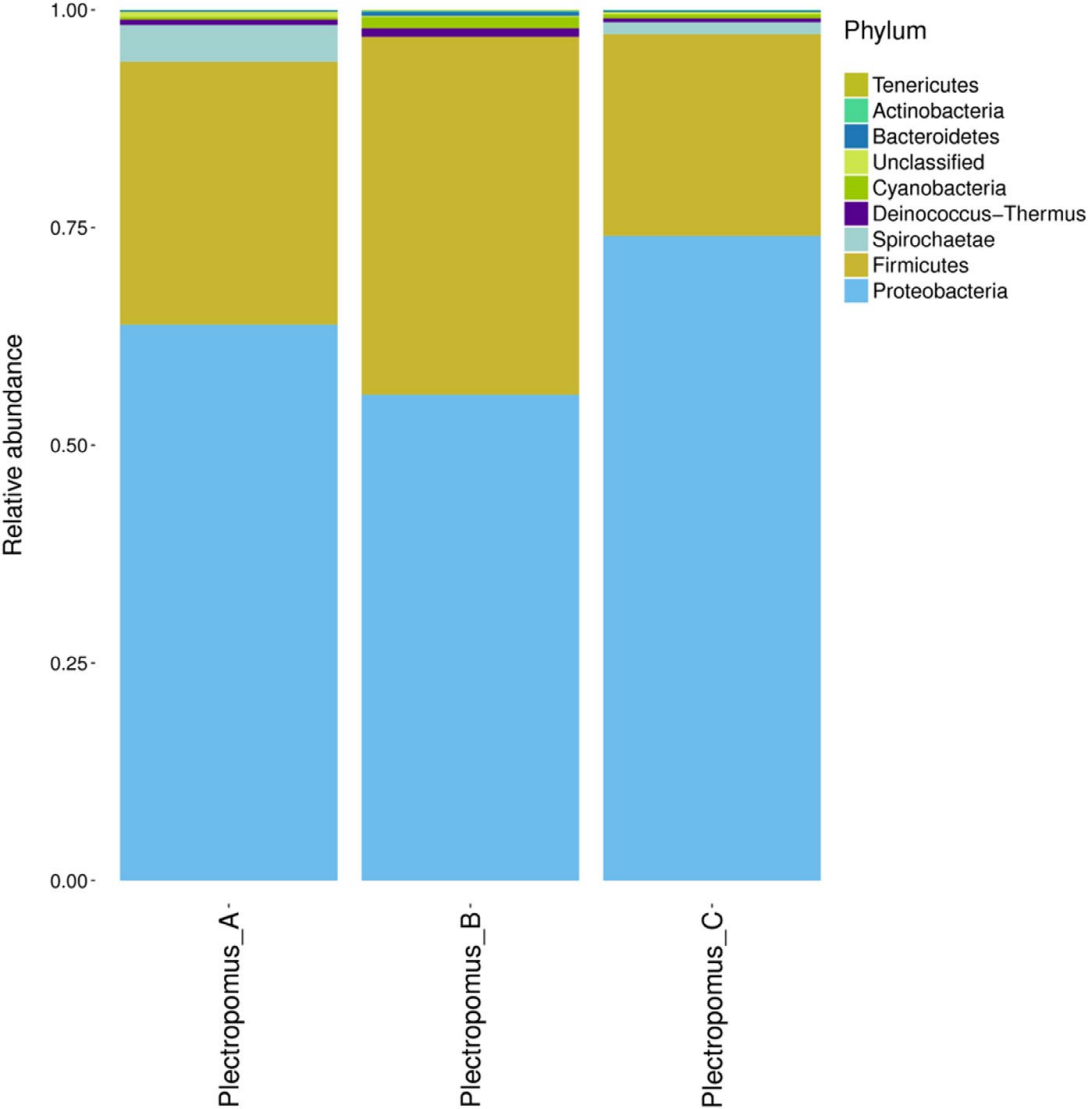
The gut bacteria communities at the phyla level of all samples in the control and enrofloxacin bathing treatment. The color‐coded bar plot showed the percentages of intestinal bacteria communities in Plectropomus_A, Plectropomus_B and Plectropomus_C at the phylum level. Plectropomus_A = control group (no enrofloxacin), Plectropomus_B = 5 mg/L enrofloxacin bathing group, Plectropomus_C = 10 mg/L enrofloxacin bathing group

A total of eight phyla were detected. *Proteobacteria* and *Firmicutes* were the dominate phyla, and there was no difference between untreated and treated groups (*p* > .05, Figure 2). The relative abundance of *Proteobacteria* in 0, 5 and 10 mg/L enrofloxacin bath groups were 63.84 ± 8.64%, 55.80 ± 1.71%, 67.39 ± 9.75% respectively, and *Firmicutes* were 35.88 ± 5.80%, 41.13 ± 1.70%, 33.16 ± 4.45%.

**FIGURE 3:**
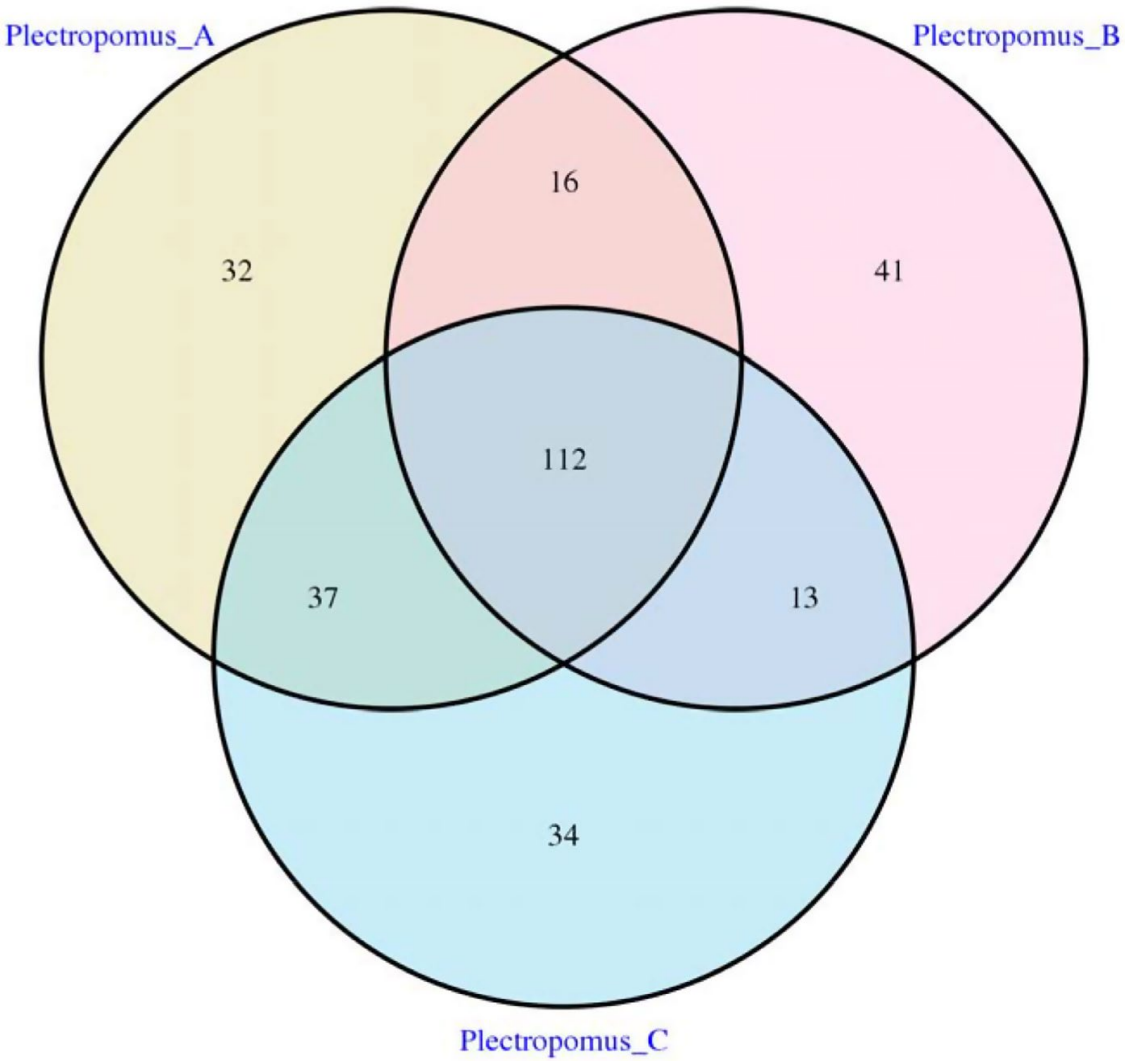
Venn diagrams showing compartmental core microbiota OTU distributions of all samples in the control and enrofloxacin bathing treatment. Plectropomus_A = control group (no enrofloxacin), Plectropomus_B = 5 mg/L enrofloxacin bathing group, Plectropomus_C = 10 mg/L enrofloxacin bathing group

**FIGURE 4:**
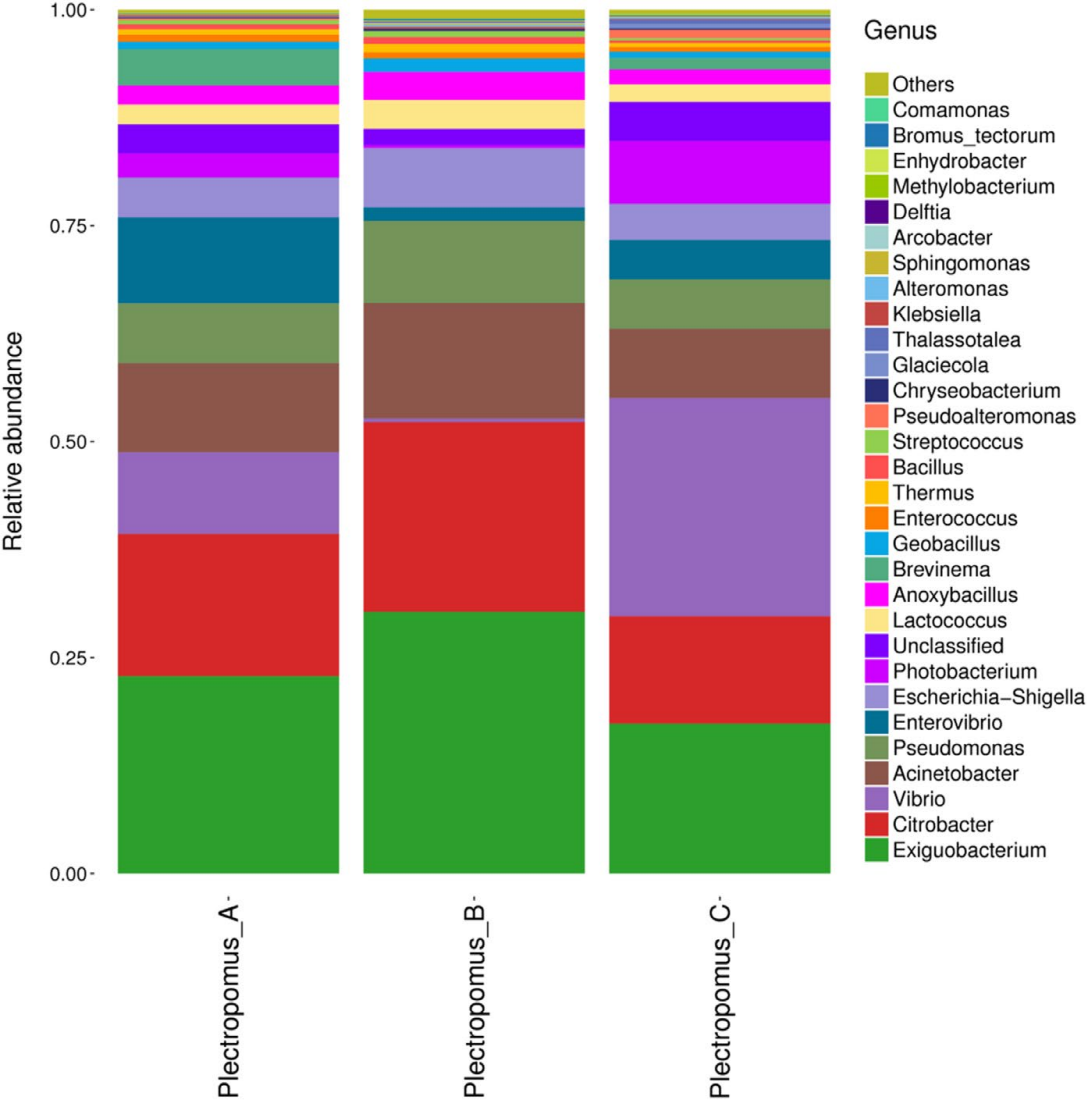
The gut bacteria communities at the genus level of all samples in the control and enrofloxacin bathing treatment. The color‐coded bar plots show the percentage of gut bacteria communities at the genus level. Plectropomus_A = control (no enrofloxacin), Plectropomus_B = 5 mg/L enrofloxacin bathing group, Plectropomus_C = 10 mg/L enrofloxacin bathing group

A total of 112 OTUs were detected as core microbiota for the digestive compartments (Figure 3), and 90 bacterial genera were detected from all samples after further analysis. *Exiguobacterium*, *Citrobacter*, *Vibrio*, *Acinetobacter* and *Pseudomonas* were the main geni in both control and enrofloxacin bathing groups. The relative abundance of the above five dominant geni were not different between the control and enrofloxacin bathing groups (*p* > .05, Figure 4). Furthermore, *Vibrio* relative abundance decreased drastically after 5 mg/L enrofloxacin bathing, while increased after 10 mg/L bathing (*p* > .05).

### 3.3 Clustering dissimilarities

**FIGURE 5:**
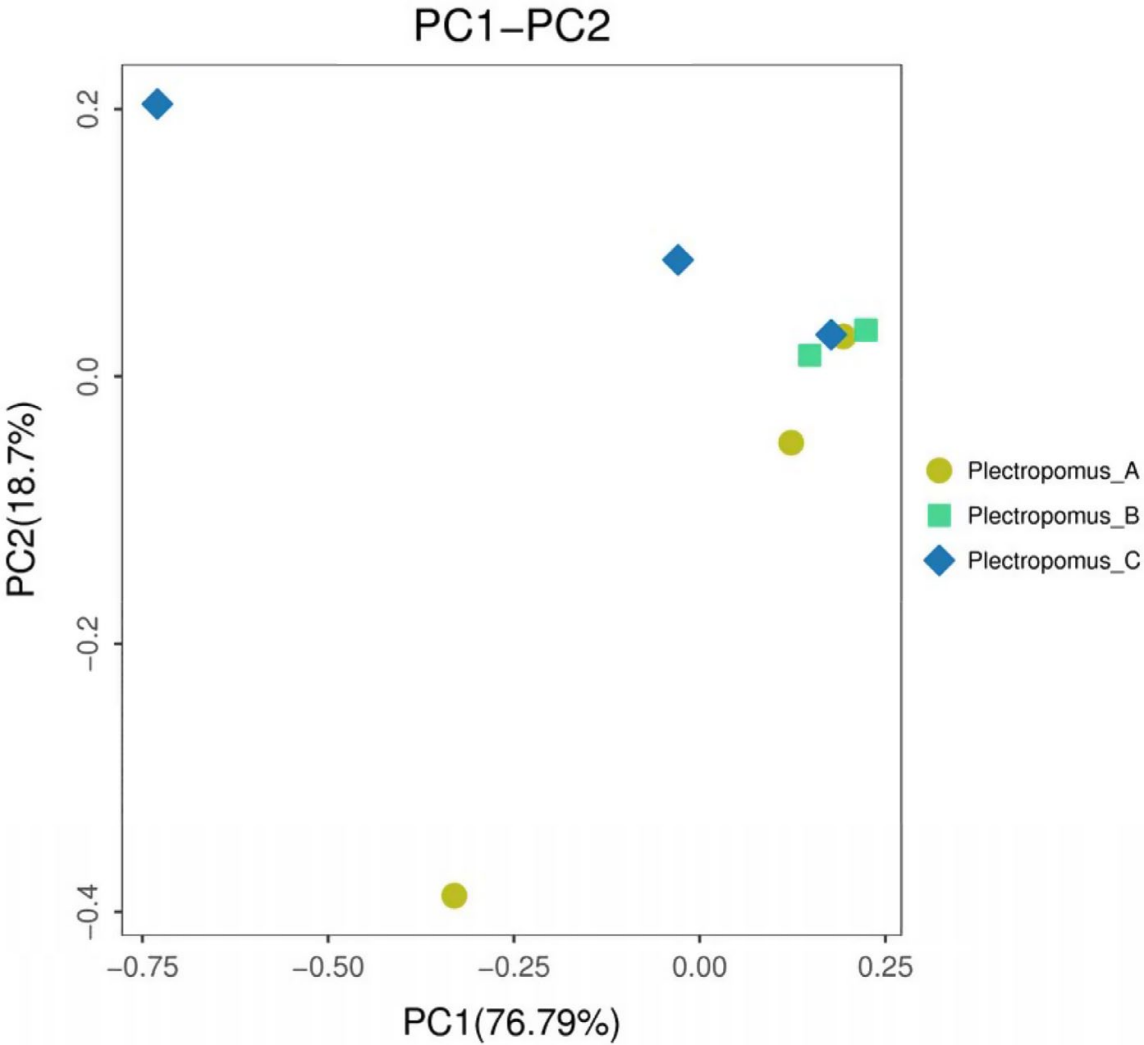
PcoA analyzed the bacteria community. The colored circles represent the bacteria in Plectropomus_A, Plectropomus_B and Plectropomus_C. Plectropomus_A = control group (no enrofloxacin), Plectropomus_B = 5 mg/L enrofloxacin bathing group, Plectropomus_C = 10 mg/L enrofloxacin bathing group

**FIGURE 6:**
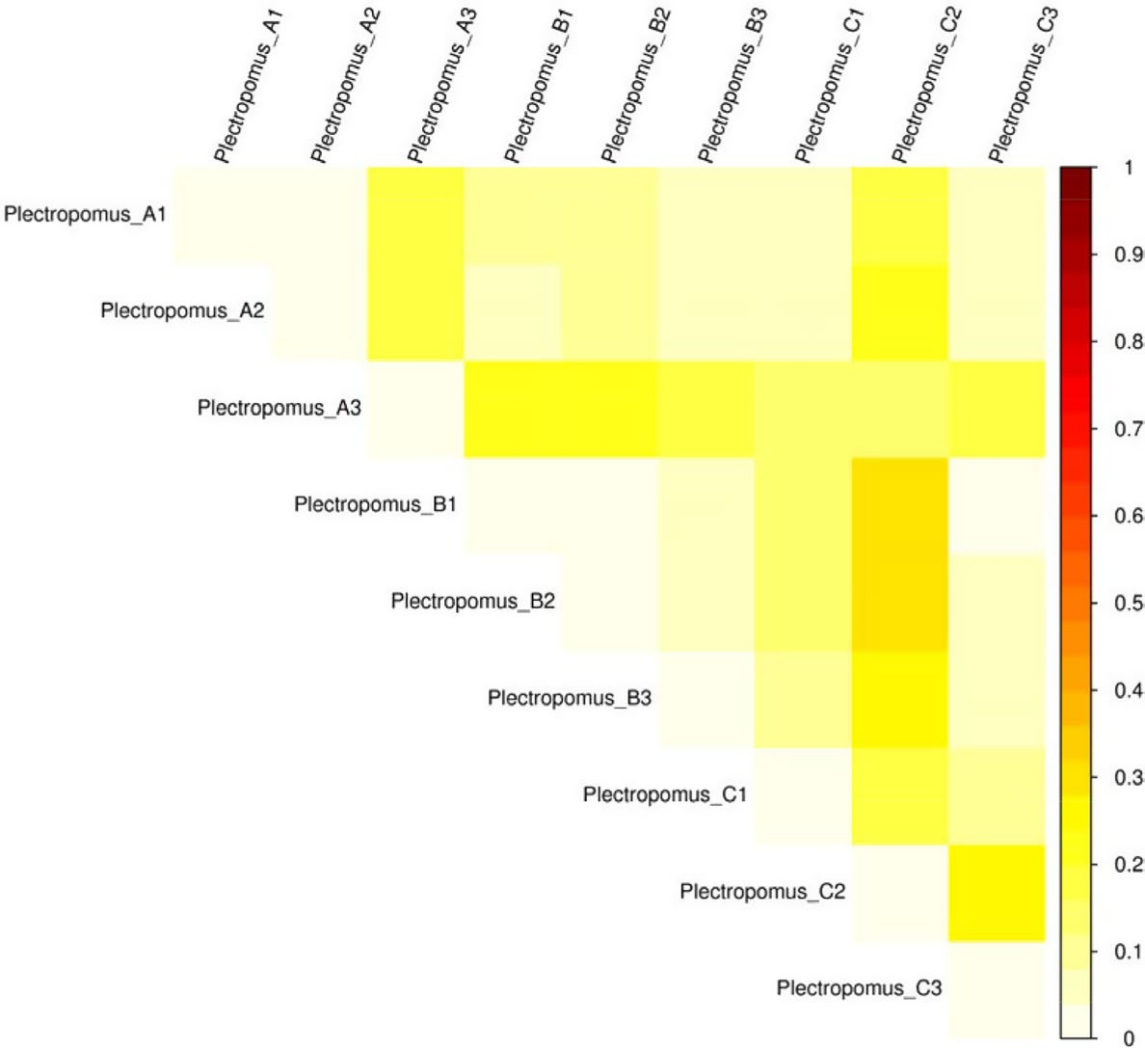
The heatmap of all samples in the control and enrofloxacin bathing treatment. The different color intensities represented the relative bacteria abundance in Plectropomus_A (A1, A2, A3), Plectropomus_B (B1, B2, B3) and Plectropomus_C (C1, C2, C3). Plectropomus_A = control group (no enrofloxacin), Plectropomus_B = 5 mg/L enrofloxacin bathing group, Plectropomus_C = 10 mg/L enrofloxacin bathing group

The principal coordinates analysis (PcoA) indicated that the bacterial community in 0, 5 and 10 mg/L enrofloxacin bathing groups clustered together, with the exception of one sample in the control and 10 mg/L enrofloxacin (Figure 5). In addition, the heatmap using weighted unifractional distances showed a similar trend to that of PCoA (Figure 6).

## 4 DISCUSSION

The gut microbiota plays an important role in the metabolism of dietary substrates and immune system modulation, having a great influence on the growth and development of the host (Moore et al., 2011; Savas, Kubilay, & Basmaz, 2005). Bacterial community composition varies with a unique core microbiome in each specific host species. *Firmicutes* and *Bacteroidetes* are the most dominant phyla in mammals (Qin et al., 2010), whereas *Proteobacteria*, *Firmicutes*, *Fusobacteria*, *Actinobacteria* and *Bacteroidetes* are the major phyla found in the intestine of carnivorous marine fish (Rückert, Palm, & Klimpel, 2008; Sevellec et al., 2014). In the present study, *Proteobacteria* and *Firmicutes* were the most common phyla in coral trout. This is in agreement with studies of the Atlantic salmon parr (Dehler, Secombes, & Martin, 2017), rainbow trout (Lyons, Turnbull, Dawson, & Crumlish, 2015) and East African cichlid (Baldo, Riera, Toomingklunderud, Albà, & Salzburger, 2015). It is speculated that *Proteobacteria* and *Firmicutes* are common gut microbes in fish, and play an important role in intestinal function. *Proteobacteria* could catabolize feedstuff components (Jumpertz et al., 2011); *Firmicutes* may be involved in energy resorption (Komaroff, 2017), and have demonstrated probiotic properties in fish (Bøgwald & Dalmo, 2014). In the present study, the dominant phyla *Proteobacteria* and *Firmicutes* in the *P. leopardus* gut were not affected by enrofloxacin bathing. It is consistent with the results of zebrafish treated by sulfamethoxazole bathing (Zhou et al., 2018).

Microbial identification is meaningful only when microbiota can be classified at the level of genus or species in relation to animal husbandry (Petrosino, Highlander, Luna, Gibbs, & Versalovic, 2009). In the present study, the major composition and relative abundance of *P. leopardus* gut bacterial communities were similar between the control and enrofloxacin bathing groups, with no difference identified between the enrofloxacin concentrations tested (5 and 10 mg/L). The five‐core microbiome geni (*Exiguobacterium*, *Citrobacter*, *Vibrio*, *Acinetobacter* and *Pseudomonas*) proportions were not altered. *Citrobacter*, *Vibrio*, *Acinetobacter*, *Pseudomonas* belong to the *Proteobacteria* while *Exiguobacterium* belongs to the *Firmicutes* genus. *Exiguobacterium* have been reported to improve the growth and survival of *Penaeus vannamei* (Sombatjinda, Wantawin, Techkarnjanaruk, Withyachumnarnkul, & Ruengjitchatchawalya, 2014), and could produce a variety of digestive enzymes aiding in nutrient resorption (Shi, Wang, & Gao, 2015). In addition, *Exiguobacterium* has been proposed for remediation of environmental pollutants (Pandey & Bhatt, 2015). However, the Vibrio relative abundance demonstrated a trend to decrease after 5 mg/L enrofloxacin bathing, but increased after 10 mg/L bathing concentrations, although this difference was not significant. The possible reason is that this genus of bacteria may be more susceptible to a low concentration of some antibiotics (Zhou et al., 2018). This observation warrants further investigation.

The alpha diversity was used for further analysis of the gut bacteria community in *P. leopardus* between the control and enrofloxacin bathing groups. Our results indicate that enrofloxacin bathing did not affect the richness and diversity of gut bacteria in *P. leopardus*. In addition, PcoA and heatmap (weighted unifrac distances) results indicate that enrofloxacin bathing treatment had little effect on gut bacterial communities of *P. leopardus*.

In summary, the gut bacteria of the coral trout after enrofloxacin bathing treatment were characterized in the present study with Illumina‐based high‐throughput sequencing. *Proteobacteria* and *Firmicutes* were the dominant phyla, and *Exiguobacterium*, *Citrobacter*, *Vibrio*, *Acinetobacter*, *Pseudomonas* were the dominant geni in *P. leopardus* gut bacteria. The gut bacteria composition (richness and diversity) was not affected by enrofloxacin bathing. Thus, enrofloxacin bathing treatment may be a safe way to prevent bacterial diseases in *P. leopardus*. The results are of importance to understand the *P. leopardus* gut bacteria community.

## CONFLICT OF INTEREST

The authors are not currently aware of any existing conflicts.

## AUTHORS CONTRIBUTION

**Xing Zheng and Gang Yu**: initiated the study conception and experimental design; **Shenjie Zhou, Jing Hu, Rui Yang**: were responsible for the acquisition of data; **Zhifeng Gu and Zhenhua Ma**: analyzed and interpreted the data; **Jian G. Qin and Zhenhua Ma**: drafted the manuscript. All authors discussed the results and contributed to the final manuscript.

## References

[vms3267-bib-0001] Austin, B. (2006). The bacterial microflora of fish, revised. Scientific World Journal, 6, 931–945. 10.1100/tsw.2006.181 16906326 PMC5917212

[vms3267-bib-0002] Baldo, L. , Riera, J. , Toomingklunderud, A. , Albà, M. , & Salzburger, W. (2015). Gut microbiota dynamics during dietary shift in Eastern African cichlid fishes. PLoS ONE, 10, e0127462. 25978452 10.1371/journal.pone.0127462 PMC4433246

[vms3267-bib-0003] Bøgwald, J. , & Dalmo, R. A. (2014). Aquaculture nutrition: Gut health, probiotics and prebiotics. UK: John Wiley & Sons Ltd.

[vms3267-bib-0004] Buchmann, K. (2015). Impact and control of protozoan parasites in maricultured fishes. Parasitology, 142, 168–177. 10.1017/S003118201300005X 23448656

[vms3267-bib-0005] Caporaso, J. G. , Kuczynski, J. , Stombaugh, J. , Bittinger, K. , Bushman, F. D. , Costello, E. K. , … Knight, R. (2010). QIIME allows analysis of high‐throughput community sequencing data. Nature Methods, 7, 335–336. 10.1038/nmeth.f.303 20383131 PMC3156573

[vms3267-bib-0006] Dehler, C. E. , Secombes, C. J. , & Martin, S. A. M. (2017). Environmental and physiological factors shape the gut microbiota of Atlantic salmon parr (*Salmo salar L.*). Aquaculture, 467, 149–157. 10.1016/j.aquaculture.2016.07.017 28111483 PMC5142738

[vms3267-bib-0007] Fang, X. , Liu, X. , Liu, W. , & Lu, C. (2012). Pharmacokinetics of enrofloxacin in allogynogenetic silver crucian carp, *Carassius auratus gibelio* . Journal of Veterinary Pharmacology & Therapeutics, 35, 397–401. 10.1111/j.1365-2885.2011.01337.x 21913940

[vms3267-bib-0008] Fang, X. , Zhou, J. , & Liu, X. (2018). Pharmacokinetics and tissue distribution of enrofloxacin after single intramuscular injection in Pacific white shrimp. Journal of Veterinary Pharmacology and Therapeutics, 41, 148–154. 10.1111/jvp.12431 28685835

[vms3267-bib-0009] Gilbert, J. , & Dupont, C. (2011). Microbial metagenomics: Beyond the genome. Annual Review of Marine Science, 3, 347–371. 10.1146/annurev-marine-120709-142811 21329209

[vms3267-bib-0010] Gu, L. , Xu, L. , Feng, J. , Su, Y. , Liu, G. , & Guo, Z. (2015). Identification and drug sensitive test of bacterial Pathogens from *Plectropomus leopardus* with tail fester disease. South China Fish Science, 11, 71–80.

[vms3267-bib-0011] Jumpertz, R. , Le, D. , Turnbaugh, P. , Trinidad, C. , Bogardus, C. , Gordon, J. , & Krakoff, J. (2011). Energy‐balance studies reveal associations between gut microbes, caloric load, and nutrient absorption in humans. American Journal of Clinical Nutrition, 94, 58–65. 10.3945/ajcn.110.010132 21543530 PMC3127503

[vms3267-bib-0012] Kang, X. , Liu, G. , Liu, Y. , Xu, Q. , Zhang, M. , & Fang, M. (2013). Transcriptome profile at different physiological stages reveals potential mode for curly fleece in chinese tan sheep. PLoS ONE, 8, e71763 10.1371/journal.pone.0071763 23990983 PMC3753335

[vms3267-bib-0013] Komaroff, A. (2017). The Microbiome and Risk for Obesity and Diabetes. JAMA, 317, 355–356. 10.1001/jama.2016.20099 28006047

[vms3267-bib-0014] Lozupone, C. , & Knight, R. (2005). UniFrac: A new phylogenetic method for comparing microbial communities. Applied and Environmental Microbiology, 71, 8228–8235. 10.1128/AEM.71.12.8228-8235.2005 16332807 PMC1317376

[vms3267-bib-0015] Lyons, P. , Turnbull, J. , Dawson, K. , & Crumlish, M. (2015). Exploring the microbial diversity of the distal intestinal lumen and mucosa of farmed rainbow trout *Oncorhynchus mykiss* (Walbaum) using next generation sequencing (NGS). Aquaculture Research, 48, 77–91.

[vms3267-bib-0016] Ma, Z. , Zhang, N. , Guo, H. , Zheng, P. , & Zhang, D. (2015). Replacement of frozen fish meat based diet with artificial diets in rearing of coral trout *Plectropomus leopardus* (Lacepede, 1802) fingerlings. Indian Journal of Fisheries, 62, 118–122.

[vms3267-bib-0017] Mekuchi, M. , Asakura, T. , Sakata, K. , Yamaguchi, T. , Teruya, K. , & Kikuchi, J. (2018). Intestinal microbiota composition is altered according to nutritional biorhythms in the leopard coral grouper (*Plectropomus leopardus*). PLoS ONE, 13, e0197256 10.1371/journal.pone.0197256 29856743 PMC5983564

[vms3267-bib-0018] Moore, A. , Christian, M. , Sommer, M. , & Gautam, D. (2011). Functional metagenomic investigations of the human intestinal microbiota. Frontiers in Microbiology, 2, 188 10.3389/fmicb.2011.00188 22022321 PMC3195301

[vms3267-bib-0019] Morgan, X. , Tickle, T. , Sokol, H. , Gevers, D. , Devaney, K. , Ward, D. , … Huttenhower, C. (2012). Dysfunction of the intestinal microbiome in inflammatory bowel disease and treatment. Genome Biology, 13, R79 10.1186/gb-2012-13-9-r79 23013615 PMC3506950

[vms3267-bib-0020] Nayak, S. (2010). Role of gastrointestinal microbiota in fish. Aquaculture Research, 41, 1553–1573. 10.1111/j.1365-2109.2010.02546.x

[vms3267-bib-0021] Pandey, N. , & Bhatt, R. (2015). Exiguobacterium mediated arsenic removal and its protective effect against arsenic induced toxicity and oxidative damage in freshwater fish, *Channa striata* . Toxicology Reports, 2, 1367–1375. 10.1016/j.toxrep.2015.10.002 28962479 PMC5598528

[vms3267-bib-0022] Paul, K. (2010). Nutrition, intestinal defence and the microbiome. Proceedings of the Nutrition Society, 69, 261–268. 20202280 10.1017/S0029665110000108

[vms3267-bib-0023] Petrosino, J. , Highlander, S. , Luna, R. , Gibbs, R. , & Versalovic, J. (2009). Metagenomic pyrosequencing and microbial identification. Clinical Chemistry, 55, 856–866. 10.1373/clinchem.2008.107565 19264858 PMC2892905

[vms3267-bib-0024] Qin, J. , Li, R. , Raes, J. , Arumugam, M. , Burgdorf, K. S. , Manichanh, C. , … Wang, J. (2010). A human gut microbial gene catalogue established by metagenomic sequencing. Nature, 464, 59–65. 10.1038/nature08821 20203603 PMC3779803

[vms3267-bib-0025] Ray, A. , Ghosh, K. , & Ringø, E. (2012). Enzyme‐producing bacteria isolated from fish gut: A review. Aquaculture Nutrition, 18, 465–492. 10.1111/j.1365-2095.2012.00943.x

[vms3267-bib-0026] Rico, A. , & Van den Brink, P. J. (2014). Probabilistic risk assessment of veterinary medicines applied to four major aquaculture species produced in Asia. Science of the Total Environment, 468–469, 630–641. 10.1016/j.scitotenv.2013.08.063 24061054

[vms3267-bib-0027] Rückert, S. , Palm, H. W. , & Klimpel, S. (2008). Parasite fauna of seabass (*Lates calcarifer*) under mariculture conditions in Lampung Bay, Indonesia. Journal of Applied Ichthyology, 24, 321–327. 10.1111/j.1439-0426.2008.01064.x

[vms3267-bib-0028] Savas, S. , Kubilay, A. , & Basmaz, N. (2005). Effect of bacterial load in feeds on intestinal microflora of seabream (*Sparus aurata*) larvae and juveniles. The Israeli Journal of Aquaculture = Bamidgeh, 57, 3–9.

[vms3267-bib-0029] Schloss, P. D. , Westcott, S. L. , Ryabin, T. , Hall, J. R. , Hartmann, M. , Hollister, E. B. , … Weber, C. F. (2009). Introducing mothur: Open‐source, platform‐independent, community‐supported software for describing and comparing microbial communities. Applied and Environmental Microbiology, 75, 7537–7541. 10.1128/AEM.01541-09 19801464 PMC2786419

[vms3267-bib-0030] Sevellec, M. , Pavey, S. A. , Boutin, S. , Filteau, M. , Derome, N. , & Bernatchez, L. (2014). Microbiome investigation in the ecological speciation context of lake whitefish (*Coregonus clupeaformis*) using next‐generation sequencing. Journal of Evolutionary Biology, 27, 1029–1046. 24773032 10.1111/jeb.12374

[vms3267-bib-0031] Shi, Z. , Wang, J. , & Gao, Q. (2015). Isolation and identification of enzyme‐producing bacteria from the digestive tract of *Epinehelus moara* in re‐circulating aquaculture systems. Journal of Fishery Sciences of China, 22, 941–949.

[vms3267-bib-0032] Sombatjinda, S. , Wantawin, C. , Techkarnjanaruk, S. , Withyachumnarnkul, B. , & Ruengjitchatchawalya, M. (2014). Water quality control in a closed re‐circulating system of Pacific white shrimp (*Penaeus vannamei*) postlarvae co‐cultured with immobilized *Spirulina* mat. Aquaculture International, 22, 1181–1195. 10.1007/s10499-013-9738-2

[vms3267-bib-0033] Suckow, M. A. , & Lamberti, G. A. (2017). Chapter 4 ‐ institutional animal care and use committee In Suckow M. A., & Stewart K. L. (Eds.), Principles of animal research for graduate and undergraduate students (pp. 65–74). Boston: Academic Press.

[vms3267-bib-0034] Tremaroli, V. , & Bäckhed, F. (2012). Functional interactions between the gut microbiota and host metabolism. Nature, 71, 242–249. 10.1038/nature11552 22972297

[vms3267-bib-0035] Turnbaugh, P. J. , Hamady, M. , Yatsunenko, T. , Cantarel, B. L. , Duncan, A. , Ley, R. E. , … Gordon, J. I. (2009). A core gut microbiome in obese and lean twins. Nature, 457, 480–484. 10.1038/nature07540 19043404 PMC2677729

[vms3267-bib-0036] Xia, J. H. , Lin, G. , Fu, G. H. , Wan, Z. Y. , Lee, M. , Wang, L. , … Yue, G. H. (2014). The intestinal microbiome of fish under starvation. BMC Genomics, 15, 266 10.1186/1471-2164-15-266 24708260 PMC4234480

[vms3267-bib-0037] Yao, X. , Xu, X. , Zhang, Z. , Ding, Z. , Song, Y. , & Cui, K. (2015). Isolation of pathogenic Listonella anguillarumfrom *Plectropomus leopardus* and its biological characterization. Periodical of Ocean University of China, 45, 39–45.

[vms3267-bib-0038] Yoseda, K. , Yamamoto, K. , Asami, K. , Chimura, M. , Hashimoto, K. , & Kosaka, S. (2008). Influence of light intensity on feeding, growth, and early survival of leopard coral grouper (*Plectropomus leopardus*) larvae under mass‐scale rearing conditions. Aquaculture, 279, 55–62. 10.1016/j.aquaculture.2008.04.002

[vms3267-bib-0039] Zhao, N. , Zhou, B. , Li, Y. , Zhang, J. , Ma, J. , & Yu, X. (2016). Effects of light color on growth, skin color, and physiological indices of juvenile *Plectropomus leopardus* in a recirculating aquaculture system. Journal of Fishery Sciences of China, 23, 976–984.

[vms3267-bib-0040] Zhou, C. , Lin, H. , Ge, X. , Niu, J. , Wang, J. , Wang, Y. , … Tan, X. (2015). The Effects of dietary soybean isoflavones on growth, innate immune responses, hepatic antioxidant abilities and disease resistance of juvenile golden pompano *Trachinotus ovatus* . Fish and Shellfish Immunology, 43, 158–166. 10.1016/j.fsi.2014.12.014 25541076

[vms3267-bib-0041] Zhou, L. , Limbu, S. M. , Shen, M. , Zhai, W. , Qiao, F. , He, A. , … Zhang, M. (2018). Environmental concentrations of antibiotics impair zebrafish gut health. Environmental Pollution, 235, 245–254. 10.1016/j.envpol.2017.12.073 29291524

